# Utilization of Crude Glycerol as a Substrate for the Production of Rhamnolipid by* Pseudomonas aeruginosa*

**DOI:** 10.1155/2016/3464509

**Published:** 2016-01-28

**Authors:** Walaa A. Eraqi, Aymen S. Yassin, Amal E. Ali, Magdy A. Amin

**Affiliations:** ^1^Department of Microbiology and Immunology, Faculty of Pharmacy, Cairo University, Cairo 11562, Egypt; ^2^Department of Pharmaceutical Microbiology, Faculty of Pharmaceutical Sciences and Pharmaceutical Industries, Future University, Cairo 11787, Egypt

## Abstract

Biosurfactants are produced by bacteria or yeast utilizing different substrates as sugars, glycerol, or oils. They have important applications in the detergent, oil, and pharmaceutical industries. Glycerol is the product of biodiesel industry and the existing glycerol market cannot accommodate the excess amounts generated; consequently, new markets for refined glycerol need to be developed. The aim of present work is to optimize the production of microbial rhamnolipid using waste glycerol. We have developed a process for the production of rhamnolipid biosurfactants using glycerol as the sole carbon source by a local* Pseudomonas aeruginosa* isolate that was obtained from an extensive screening program. A factorial design was applied with the goal of optimizing the rhamnolipid production. The highest production yield was obtained after 2 days when cells were grown in minimal salt media at pH 6, containing 1% (v/v) glycerol and 2% (w/v) sodium nitrate as nitrogen source, at 37°C and at 180 rpm, and reached 2.164 g/L after 54 hours (0.04 g/L h). Analysis of the produced rhamnolipids by TLC, HPLC, and FTIR confirmed the nature of the biosurfactant as monorhamnolipid. Glycerol can serve as a source for the production of rhamnolipid from microbial isolates providing a cheap and reliable substrate.

## 1. Introduction

Biosurfactants are surface active compounds produced by microorganisms. They are also known as microbial surface active compounds (SACs). There are many types of biosurfactants based on their chemical composition such as glycolipids, lipopolysaccharides, oligosaccharides, and lipopeptides that have been reported to be produced by diverse bacterial genera [1, 2]. The best-studied biosurfactants are glycolipids, such as rhamnolipids produced by* Pseudomonas*, sophorolipids produced by different species of the yeast* Candida* (formerly* Torulopsis*), cell-bound trehalose lipids produced by* Rhodococcus* and other* Actinomycetes*, and a variety of structurally different lipopeptides produced by several* Bacillus* species [3–5].

Biosurfactants are receiving an increasing attention due to their potential commercial and environmental applications as substitutes for synthetic surfactants. They exhibit high surfactant and emulsifying activities and they are stable under extreme chemicophysical conditions. Biosurfactants possess environmentally friendly characteristics such as low toxicity and high biodegradability [6–8]. Accordingly, public acceptance is higher for microbial SACs than synthetic surfactants [9]. Biosurfactants have been used to enhance contaminant removal in soil and water [10, 11]. They have also been used in chemicophysical processes designed to remediate hydrocarbon or heavy metal contaminated sites [12]. Due to their heterogeneity, microbial SACs display a broad range of potential applications in oil, agricultural, cosmetic, and food industries [9].

Despite the advantages and potential applicability of these biological compounds, the success of biosurfactants depends on the economy of the production process and the use of low cost raw materials which account for the 10–30% of the overall costs [13, 14]. The utilization of waste glycerol is becoming very important, because the amount of waste has been increasing year by year through the increasing production of biodiesel and other oleochemicals [15]. On the other hand, glycerol is successfully used as the water-soluble carbon source for different microbial productions [16, 17].

The aim of the present work is to optimize the production of rhamnolipids by* Pseudomonas aeruginosa* grown on waste glycerol as a substrate, by studying the effects of contributing factors individually and collectively and identifying the most appropriate production conditions, and to characterize the produced rhamnolipids.

## 2. Material and Methods

### 2.1. Enrichment and Isolation Procedure

A total of 20 different strains were tested for rhamnolipid production after being isolated from different samples obtained from oil polluted surfaces and machines at different gas stations (Giza, Egypt). Enrichment cultures were prepared in minimal salt medium (MSM) supplemented with hydrophobic source as sole carbon source (olive oil). Each sample was incubated for 15 days with agitation 180 rpm at 30°C; an aliquot of each culture was serially diluted and streaked on brain heart agar plates. Colonies with different morphologies were isolated by repeated streaking on the same medium. Bacterial suspension for each isolate was prepared in 50 mL MSM and incubated overnight at 37°C with shaking at 180 rpm. After adjusting the OD to 0.5 McFarland, 1 mL was inoculated in 250 mL flasks containing 100 mL MSM and left at 180 rpm at 30°C for 5 days. An aliquot of 10 mL was taken from each flask, centrifuged at 6,800 ×g rpm for 15 min to remove bacterial cells, and the supernatant was screened for surface activity through three different tests. In addition, 20 previously identified environmental isolates of* P. aeruginosa* were used for comparison.

### 2.2. Testing for Surface Activity

#### 2.2.1. Oil Displacement Test

A volume of 15 *μ*L of crude oil was placed on the surface of 40 mL of distilled water placed in a petri dish, and a supernatant of 10 *μ*L of each culture was gently placed on the surface of the oil film. Diameter of the clear halo viewed under visible light was measured after 30 s [18].

#### 2.2.2. Emulsification Activity

A volume of 3 mL of xylene was vortexed with 3 mL of supernatant for 2 min and allowed to settle for 24 h, and then the emulsification index (*E*_24_) was estimated as follows:

(1) $$\begin{array}{c} E_{24}=\frac{h_{emulsion}}{h_{total}}\times 100\%, \end{array}$$

where *h*_emulsion_ is the height of emulsion layer and *h*_total_ is the height of total liquid column.

#### 2.2.3. Mineral Salt-CTAB-Methylene Blue Agar Plate Method

Shallow wells were cut into the surface of the indicator plates with the heated point of a 10 mL glass pipette. Ten microliters of the appropriate culture was placed into each well. The plates were then incubated at the proper temperature and checked periodically over a 24 to 48 h time period. A positive reaction for rhamnolipid was the formation of a purple-blue haze with a sharply defined edge around the culture well. After incubation, plates were placed at 4°C for a few days. This caused positive reactions to darken significantly and made it possible to visualize weak positive reactions that were not apparent upon initial inspection [19].

### 2.3. Time Course of Rhamnolipid Production Using Glucose and/or Glycerol as Sole Carbon Source

Fermentation was carried out in MSM at 37°C with shaking at 180 rpm for different periods: 1, 2, 3, 4, and 5 days. Crude glycerol used contained 50% to 60% standard glycerol and was originally obtained from biodiesel production (Tagadod Company, Egypt). Glycerol content in fermentation media was calculated in terms of standard glycerol equivalent.

### 2.4. Optimization of Rhamnolipid Production from Glycerol

Optimization of various conditions for rhamnolipid production from glycerol was carried out for the promising isolate. Different factors were studied including nitrogen source (inorganic sources: sodium nitrate, ammonium nitrate and ammonium sulphate, and organic source: urea), different glycerol concentrations (0.5%, 1%, 2%, 3%, and 4%), pH values (4, 6, 7, 8, and 10), incubation temperature (30°C, 37°C, and 42°C), and effect of shaking rate (static condition: 100 rpm, 180 rpm, and 250 rpm).

### 2.5. Quantitative Determination of Rhamnolipid

A volume of 0.5 mL of culture supernatant was extracted twice with 1 mL of diethyl ether. The ether fractions were pooled and evaporated to dryness and reconstituted in 0.5 mL H_2_O. Samples were diluted 1/10 in a solution containing 0.19% orcinol in 53% H_2_SO_4_. The sample was then placed in boiling water for 30 min and cooled at room temperature for 15 min, and the absorbance (*A*_421_) was measured [20]. The rhamnolipid concentrations were calculated from standard curves prepared with l-rhamnose and expressed as rhamnose equivalents (RE) (mg/L).

### 2.6. Experimental Factorial Design

Full factorial two-level design (2^4^) was done with a total of 16 runs to evaluate the influence of independent factors and the possible interactions between them against the dependent variable of the rhamnolipid concentration. The statistical software package Minitab 16, USA, was used to design the experiment and regression analysis of experimental data and in plotting relationship between variables. The chosen variables in the two-level forms were temperature (42°C or 37°C), rpm (100 or 180), pH value (6 or 7), and glycerol concentration (1% or 0.5%). High and low level of each factor were selected according to the results of the previous experiments based on the conventional change of one factor at a time. The main effects of parameters on rhamnolipid production were estimated by subtracting the mean responses of variables at their lower levels from their corresponding higher levels and dividing by the total number of experimental runs. The quality of fit of the first-order model was tested and the parameters with statistically significant effects and interactions were identified using Fisher's test for the analysis of variance (ANOVA).

### 2.7. Isolation and Partial Purification of the Crude Rhamnolipid

Bacterial cells were first removed from the culture broth by centrifugation at 6,800 ×g for 15 min and then the supernatant was acidified using conc. HCl to pH 2.0 and kept at 4°C overnight. Rhamnolipids were then pelleted by centrifugation at 12,000 ×g for 20 min and transferred to a separating funnel, extracted three times with a chloroform-ethanol (2 : 1 v/v) mixture with vigorous shaking, leaving the two layers to be separated in the funnel. The organic layer was then evaporated in air leaving behind relatively pure rhamnolipids having oil-like appearance [21].

### 2.8. Characterization of Partially Purified Rhamnolipid

#### 2.8.1. Thin Layer Chromatography

Previously purified rhamnolipids were dissolved in chloroform and 10 *μ*L was applied onto a TLC plate (silica gel 60, Sigma, USA) at a point of origin near the bottom of the plate. Once dried, the plate was developed in solvent system of chloroform : methanol : acetic acid (6.5 : 1.5 : 0.2, v/v/v) [22]. When developed, the plate was removed and allowed to air-dry and then it was evenly sprayed with anthrone reagent, prepared by mixing 63 mL of sulfuric acid, 25 mL of water, and 0.125 g of anthrone under ice conditions, and placed in an oven at 110°C for 20 min. Upon visualization, the spot nearer the point of origin corresponded to the dirhamnolipids, while the spot further from the point of origin represented the monorhamnolipids.

#### 2.8.2. Fourier Transform Infrared (FTIR) Spectrophotometer

The partially purified pellet was dissolved in water and the IR spectra were recorded on a FTIR spectrometer in the 4000–400 cm^−1^ spectral region at a resolution of 2 cm^−1^. Comparison was made to standard rhamnolipid R-95 (Sigma, USA).

#### 2.8.3. High Performance Liquid Chromatography (HPLC)

The partially purified pellet was redissolved in 1.5 mL of acetonitrile (a content of approximately 0.1–1 mM was achieved by appropriate dilution), 1 mL of this solution was mixed with 200 *μ*L of the derivatization agent (1 : 1 (v/v) solution of 40 mM 4-bromophenacyl bromide and 20 mM triethylamine in acetonitrile), and derivatization reaction took place at 60°C for 90 min. Subsequently, the rhamnolipids were separated in a reverse phase C18 column (Supelcosil LC-18, Supelco/Sigma-Aldrich cooperation, Bellefonte, PA, USA) on an HPLC device (Young Lin Y9100, Korea) with a linear gradient of acetonitrile-water and finally detected by a UV-detector at 265 nm [23].

## 3. Results

### 3.1. Isolation and Testing of Strains

A total of 20 microbial strains were isolated that showed positive biosurfactant activity using OST, *E*_24_, and blood hemolysis tests. Preliminary identification of the isolates indicated the diversity of the biosurfactant producing strains as they included gram negative rods of* Pseudomonas* and* Klebsiella* species and* Bacillus* and* Candida*. The isolate used in this study displayed the highest emulsification index and oil spreading activity among the whole collection and was identified as* P. aeruginosa* by 20 NE API system and was labeled as* P. aeruginosa* WAE. Accordingly, biosurfactant production of this isolate was compared with that of a collection of 20 previously identified environmental isolates of* P. aeruginosa* using CTAB and the results showed that it still had the highest activity. Therefore, the strain was selected for further investigations. The 16S rDNA gene sequencing showed the highest similarity (89%) with* P. aeruginosa* DSM50071 16S rDNA sequence available at the NCBI database using BLAST server (http://blast.ncbi.nlm.nih.gov/).

### 3.2. Examining the Role of Different Carbon Source

**Figure 1:**
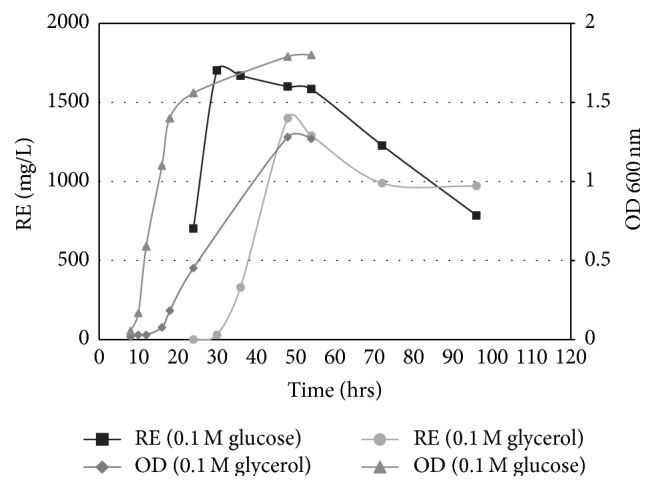
Comparison between using 0.1 M glycerol and 0.1 M glucose as the sole carbon source and their effects on OD and rhamnolipid production expressed as rhamnose equivalent (RE).

The production of rhamnolipid by* P. aeruginosa* WAE was compared using glucose or glycerol as the sole carbon source. The produced concentration of rhamnolipid was 1700 mg/L and 1400 mg/L when using glucose or glycerol, respectively, indicating that glycerol can provide the fermentation medium with an adequate carbon source (Figure 1).

### 3.3. Optimization of Rhamnolipid Production from Glycerol by* P. aeruginosa* WAE

#### 3.3.1. Effect of Glycerol Concentration

The effect of different glycerol concentration (0.5%, 1%, 2%, 3%, and 4%) on rhamnolipid production was evaluated; fermentation was done in MSM supplemented with 2% NaNO_3_, pH 7, 180 rpm, and 37°C. The results show that the highest levels of rhamnolipids (1350–1400 mg/L) were achieved when using 0.5% or 1% glycerol after approximately 48 hours. Raising the concentration of glycerol to 2%-3% led to a slight increase in the yield up to 1450 mg/L but after 96 hours. Additional increase in glycerol concentration had an inhibitory effect as the production of rhamnolipid diminished.

#### 3.3.2. Effect of Nitrogen Source

**Figure 2:**
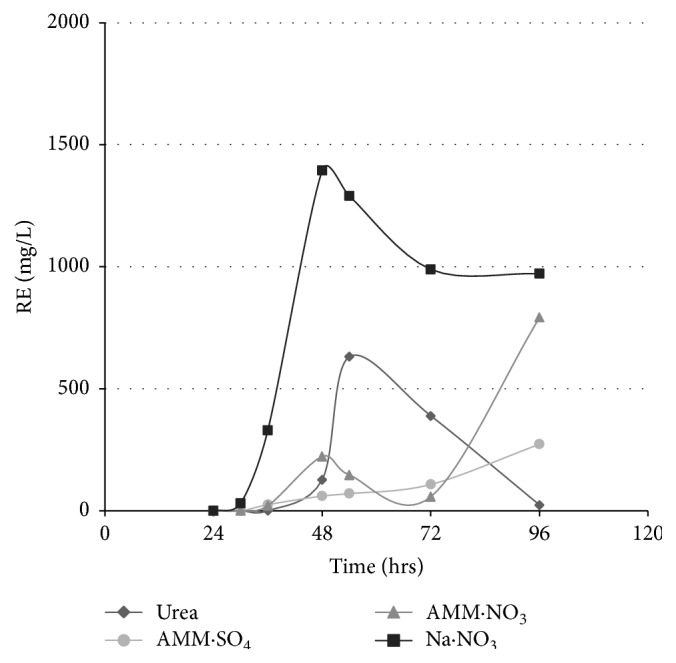
Effect of different nitrogen sources on rhamnolipid production expressed as rhamnose equivalent (RE).

The effects of different nitrogen sources including sodium nitrate, ammonium nitrate, ammonium sulphate (inorganic nitrogen sources), and urea (organic nitrogen source) on rhamnolipid production were tested in MSM of pH 7 with 1% glycerol incubated at 37°C and 180 rpm. The highest levels were observed when using sodium nitrate after 48 hours (1359 mg/L), followed by ammonium nitrate after 4 days (792 mg/L) and then urea in case of urea after 54 hours (630 mg/L). The lowest production levels were observed when using ammonium sulphate even if after 96 hours (Figure 2).

#### 3.3.3. Effect of Temperature

**Figure 3:**
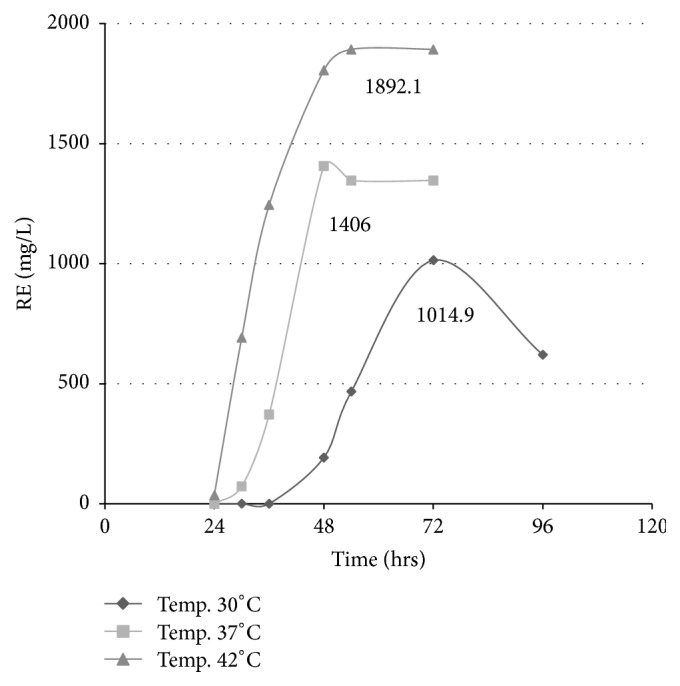
Effect of different temperatures on rhamnolipid production expressed as rhamnose equivalent (RE).

Fermentation was done in MSM pH 7 supplemented with 1% glycerol and 2% NaNO_3_ at 180 rpm and different temperatures (30°C, 37°C, and 42°C) were tested. The highest rhamnolipid concentration 1892 mg/mL was reached when fermentation was done at 42°C; lower values of 1402 mg/L and 1015 mg/L were obtained at 37°C and 30°C, respectively (Figure 3).

#### 3.3.4. Shaking Rate (Aeration)

**Figure 4:**
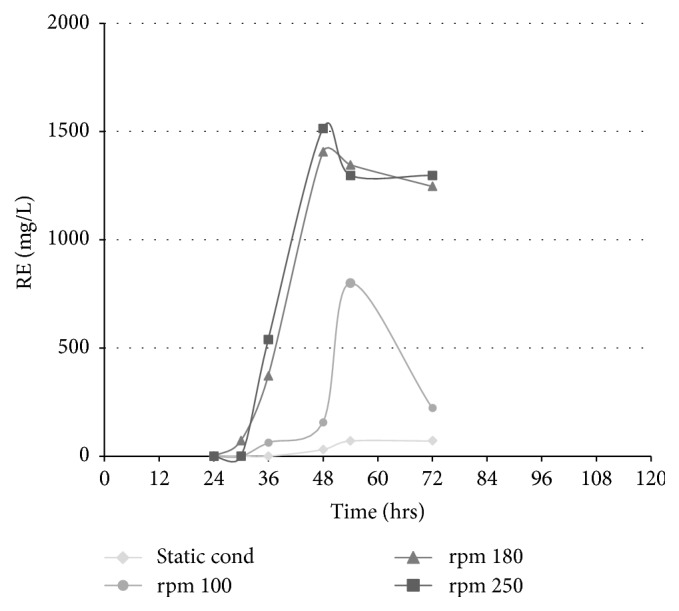
Effect of different shaking conditions on rhamnolipid production expressed as rhamnose equivalent (RE).

Fermentation was done in MSM pH 7 supplemented with 1% glycerol and 2% NaNO_3_ at 37°C and different shaking rates (100, 180, and 250) were tested and fermentation under static condition was tested. The results showed that fermentation at either 180 or 250 rpm gave almost relatively comparable productivity (1514 mg/mL and 1406 mg/mL, resp.) after 48 hours, while there was significant decrease in the rhamnolipid concentration to 800 mg/mL by decreasing the shaking rate to 100 rpm and 71 mg/mL at static condition (Figure 4).

#### 3.3.5. Effect of pH

Fermentation was done in MSM supplemented with 1% glycerol and 2% NaNO_3_ at 180 rpm and 37°C and different pH values (4, 6, 8, and 10) were tested. The highest yield was obtained when fermentation was done at initial pH of 6 to reach rhamnolipid concentration of 1977 mg/L at 48 hours. Lower concentration was obtained at pH 7 and pH 8 (1406 mg/L and 818 mg/L, resp.). The concentration of rhamnolipids was undetectable at both pH 10 and pH 4.

#### 3.3.6. Experimental Factorial Design

The effect of four variables (glycerol conc., temperature, rate of agitation, and pH) and the possible interactions between them were investigated by constructing a factorial design set of experiments using Minitab version 16 and two values for each variable, a higher one and a lower one. The higher level and lower level for each variable were temperature (42°C or 37°C), rpm (100 or 180), pH value (6 or 7), and glycerol concentration (1% or 0.5%). The different combinations of variables for all experiments and the corresponding rhamnolipid concentration are shown in Table 1. All runs were done at 54-hour interval. Highest rhamnolipid concentration was obtained in the 15th run at pH 6, 180 rpm, glycerol conc. 1%, and temp. 37°C where all variables were in their higher level except the temperature which was in the lower level and reached 2164 mg/L after 54 hours.

**Figure 5:**
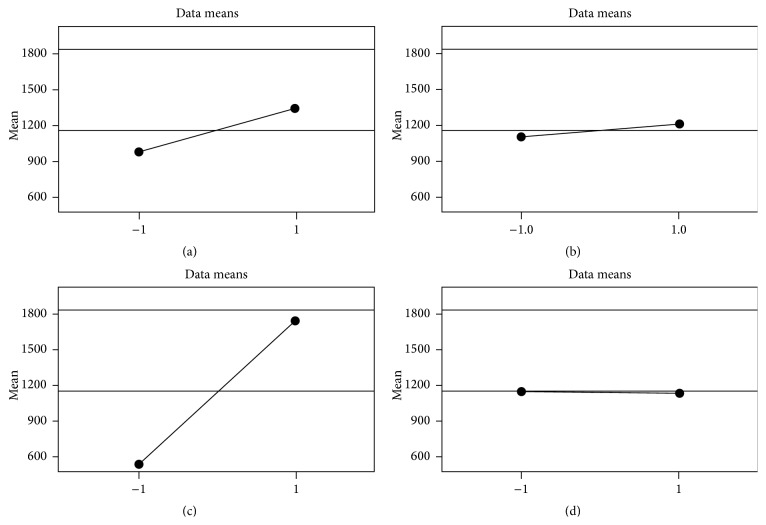
Main effect of variables: (a) pH value, (b) glycerol concentration, (c) rpm value (180 or 100), and (d) temperature (42°C or 37°C).

Main effect plot showed the positive effect of rpm, pH value, and glycerol concentration observed as an increase in the slope line between their +1 and −1 levels. Although the plot did not show great effect for glycerol concentration as a variable, its effect was then proved to be significant by *t*-test. In case of temperature, no significant effect was found (Figure 5).

The adequacy of the model was tested and the parameters with statistically significant effects or interactions were identified using one-way ANOVA. Both *T*-value and *P* value statistical parameters were used to confirm the significance of the factors. The results showed that pH value, glycerol concentration, and rpm all had significant effect (*P* ≤ 0.05). The model determination coefficient (*R* = 0.99) suggested that the fitted model could explain 99% of the total variation, implying a satisfactory representation of the process by the model.

The mathematical expression between the four variables for rhamnolipid production is given in an equation which allows the prediction of the response in further future experiments. The mathematical formula is originally *Y* = *a* + *bx*, where “*Y*” is the rhamnolipid concentration, “*a*” is the constant, and *x* is the concentration of the variable. The variables are represented as follows: *A*: pH value (6 or 7), *B*: glycerol concentration (1% or 0.5%), *C*: rpm value (180 or 100), and *D*: temperature (42°C or 37°C). Consider

(2) Rhamnolipid  Concentration  Predicted=1154.83+178.61A+55.35B+610.61C+84.89A×B+77.25A×C+134.61B×C+60.02B×D−44.74C×D−32.82A×B×C+26.04A×B×D−38.35A×C×D−59.18A×B×C×D.

### 3.4. Characterization of Partially Purified Rhamnolipid

#### 3.4.1. Thin Layer Chromatography

When the partially purified product was separated on TLC plate alongside with a sample of commercially available rhamnolipid, as an authentic control, the product from the authentic control sample on the paper chromatogram showed two spots while that from* P. aeruginosa* WAE showed only one single brown spot which was equidistant with the higher spot (monorhamnolipid) of the control sample (95% rhamnolipid).

#### 3.4.2. Fourier Transform Infrared (FTIR) Spectrophotometer

**Figure 6:**
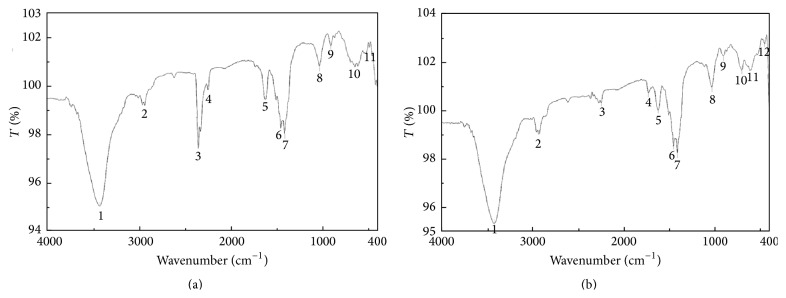
FTIR spectrum of (a) standard rhamnolipid and (b) rhamnolipid produced by* Pseudomonas aeruginosa*.

Investigation of structural features of the partially purified product included using FTIR spectroscopy, and it revealed the identity of fingerprint region with that of the standard rhamnolipid (Figure 6).  Table 2 shows the patterns revealed by the following groups: free -OH stretching at 3430 cm^−1^, the C-H stretching vibrations of CH_2_ and CH_3_ groups at 2938 cm^−1^, C=O from ester and carboxylic groups that were observed at 1739 cm^−1^ and 1629 cm^−1^, respectively, C-O stretch at 1042 cm^−1^, and C-O-C stretching in the rhamnose at 1042 cm^−1^.

#### 3.4.3. High Performance Liquid Chromatography

**Figure 7:**
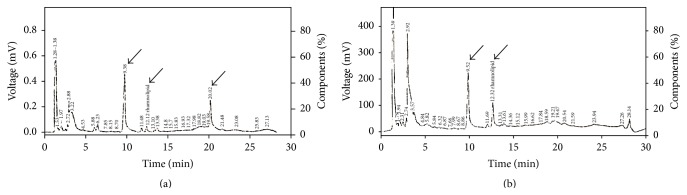
Chromatograms of (a) standard rhamnolipid and (b) rhamnolipid produced by* Pseudomonas aeruginosa* WAE. The arrows refer to monorhamnolipid (9.38, 12.12, in the standard sample, and 9.52, 12.32 in* Pseudomonas aeruginosa* WAE) and dirhamnolipids (20.02, in standard sample).

Two peaks in the chromatogram of the standard rhamnolipid at retention time (9.38 and 12.12 min) were assigned to monorhamnolipids and one peak at 20.02 min was assigned to dirhamnolipid (Figure 7). The chromatogram of the tested sample also had the two peaks at (9.52 and 12.32 min), confirming the presence of monorhamnolipid compound and no peaks equivalent to dirhamnolipids were present. The presence of other small peaks at 11.4, 13.3, and 20.2 min showed the possibility of the presence of different rhamnolipid isomers in the culture broth. The two sharp peaks appearing at retention times (1.3 and 2.9 min) in the chromatograms of the standard and the sample were assigned to the derivatizing agents 2-bromoacetophenone and triethylamine as both appeared in a blank control with no sample or standard (data not shown).

## 4. Discussion

Microbial SACs are receiving an increasing attention due to their potential commercial applications as substitutes for synthetic surfactants. Our goal in this work is to optimize the production of rhamnolipids from a local strain grown on waste glycerol obtained from biodiesel industry as a substrate.

For the screening of biosurfactant producing microbes, enrichment cultures utilizing hydrophobic compounds as the sole carbon source are usually applied. Enrichment was done using minimal salt media supplemented with 2% olive oil. Isolates were collected from air, oil polluted surfaces, and hydrocarbon polluted soil.

For efficient detection of potential biosurfactant producers, combinations of various screening methods were required. Oil spreading test was recommended to be the second most suitable method after surface tension measurement in primary screening [24]. The oil spreading technique had a larger dynamic range than surface tension. It was also easy to perform and to standardize and was less time-consuming than surface tension measurements. One of the isolates, later identified as* P. aeruginosa* WAE, had the most potent emulsification activity and was chosen for further investigation. The high productivity of rhamnolipid by the selected isolate compared to all other isolates was further confirmed by the CTAB assay.

Substitution of glucose in the culture medium by glycerol as a sole carbon source had no dramatic effect on the growth as well as rhamnolipid productivity. There was a little delay in the growth in case of using glycerol and since rhamnolipids are typical secondary metabolites, a corresponding delay in the rhamnolipid production was also observed. Adjustment of culture aeration, temperature, and pH increased the rhamnolipid production from glycerol to reach about 2 g/L which exceeded the level of production from glucose, providing strong evidence that waste glycerol from biodiesel industry could be economically utilized as a substrate for rhamnolipid production.

Upon studying the effect of variable concentrations of glycerol, as a sole carbon source, our results showed that the maximum yield of rhamnolipid (g/L) was reached using glycerol concentrations from 1 to 3%. Increasing glycerol concentration above 3% was accompanied by an inhibitory effect on microbial growth and the production of biosurfactants. This inhibitory effect was ascribed to problems linked to the solubility of glycerol and the difficulty of the bacterium to gain access to the nutrients in the culture medium. Similar results were observed in other studies [25].

Among different tested nitrogen sources, sodium nitrate was selected as it gave the highest yield of rhamnolipid compared to other nitrogen sources as previously observed [25, 26]. Studies have indicated a direct relationship between increased glutamine synthetase activity and enhanced biosurfactant production in* Pseudomonas aeruginosa* grown in nitrate and proteose peptone media and indicated that high levels of NH^+4^ or glutamine reduce the production of rhamnolipids, and this is associated with glutamine synthase activity [27].

Several pH values were examined and maximum yield of rhamnolipid was obtained at pH 6. Decreasing the pH to 4 resulted in undetectable amount of rhamnolipid. This could be explained by the presence of rhamnolipid under acidic conditions in their protonated form and therefore they become less soluble in water [28, 29]. An agitation rate of 250 rpm gave the highest yield of rhamnolipid. On the other hand, production decreased by lowering the agitation rate to 100 rpm. Such an increased yield at a high agitation rate could be explained due to a preference in microaerobic conditions for growth. A similar was observed previously with* P. aeruginosa* PAO1, as the high concentration of oxygen in the growth medium appears to exert a stress upon the organism, leading to a reduced growth rate, a longer lag phase, and a greater release of proteins per gram of biomass formed [30].

Increasing the inoculum size did not show any change in the rate of rhamnolipid production; this could be explained by the fact that rhamnolipid is a secondary metabolite and its production is related to a quorum sensing system, which is activated in high cell densities, so it represents a direct link between high cell population and rhamnolipid production activation phase [31, 32]. In addition, most quorum sensing regulated genes are not induced before the stationary growth phase [33].

The variables chosen to evaluate the significance of their effect and the possible interactions on rhamnolipid production were glycerol concentration, pH value, agitation rate, and temperature. High and low level of each factor were selected according to the results of the experiments that were based on the conventional change of one factor at a time. These particular four variables were chosen when tested individually; each variable could provide high rhamnolipid yield at the two values representing the high and low value of each. Consequently, our aim was to fully optimize the whole operation among these four variables and this was observed in run number 15. First-order model that can predict the rhamnolipid productivity (dependent variable) as a function of the independent variables was then constructed. Fischer's *F*-test showed a value which was much greater than that of the *F* tabulated and that demonstrates that the model terms are significant. Our maximum yield was 2.164 g/L after 54 hours (0.04 g/L h). Previous production levels of well characterized strains as* P. aeruginosa* PAO1 were 39 g/L grown on sunflower oil on a large scale bioreactor or 2.2 g/L on small scale level which is comparable to our strain [34].

Analysis of the purified product through TLC plates indicated the production of only monorhamnolipid in the culture media of* P. aeruginosa* WAE by the presence of only one spot which was equidistant with the higher spot of the standard one. Investigation of structural features of the partially purified product included using FTIR spectroscopy and revealed the identity of fingerprint region with that of the standard rhamnolipid. HPLC analysis confirmed the presence of the monorhamnolipid compound. The presence of other small peaks indicated the possibility of the presence of different rhamnolipid isomers in the culture broth. The fact that our strain* P. aeruginosa* WAE produces only monorhamnolipid indicates that it might be in the same clade as* P. aeruginosa* PA7 which was previously sequenced and found to lack* rhlC* gene responsible for conversion of monorhamnolipid to dirhamnolipid [35].

## 5. Conclusions

Waste glycerol can provide a cheap yet valuable source for the production of rhamnolipids from microbial isolates. Careful adjustment of individual factors followed by a factorial design can lead to the optimization of production conditions to adequate levels.

## Figures and Tables

**Table 1:** The experimental conditions for all experiments and the corresponding rhamnolipid yield.

Run	pH	Glycerol conc.	rpm	Temp.	RL. conc. mg/L
1	−1	−1	−1	−1	430.1
2	−1	−1	−1	1	510.0
3	−1	−1	1	−1	1295.4
4	−1	−1	1	1	1108.4
5	−1	1	−1	−1	428.4
6	−1	1	−1	1	402.9
7	−1	1	1	−1	1722.1
8	−1	1	1	1	1912.4
9	1	−1	−1	−1	853.4
10	1	−1	−1	1	700.4
11	1	−1	1	−1	2075.7
12	1	−1	1	1	1822.4
13	1	1	−1	−1	302.6
14	1	1	−1	1	725.9
15	1	1	1	−1	2164.1
16	1	1	1	1	2023.0

**Table 2:** Characteristic absorption of rhamnolipid functional groups in infrared analysis.

Peak number	Characteristic absorption (cm^−1^)	Functional group
1	3430	Strong and broad bands of the hydroxyl group free (-OH) stretch due to hydrogen bonding
2	2938	The aliphatic bonds CH_3_, CH_2_, and C-H stretching
3	2254	Unresolved
4	1736	Carbonyl (C=O) stretching (ester)
5	1629	Carbonyl (C=O) stretching (acidic)
6	1462	The aliphatic C-H bending
7	1421	The presence of *carboxylic acid functional group* in the molecule was *confirmed* by the bending of the hydroxyl (O-H) at 1421 cm^−1^
8	1042	C-O-C stretching in the rhamnose
9	915	Fingerprint region and it is identical with that of the standard rhamnolipid
10	703	
11	614	
12	450	

## References

[1] Banat I. M., Franzetti A., Gandolfi I. (2010). Microbial biosurfactants production, applications and future potential. *Applied Microbiology and Biotechnology*.

[2] Franzetti A., Gandolfi I., Bestetti G., Smyth T. J. P., Banat I. M. (2010). Production and applications of trehalose lipid biosurfactants. *European Journal of Lipid Science and Technology*.

[3] Lang S., Philp J. C. (1998). Surface-active lipids in *rhodococci*. *Antonie van Leeuwenhoek*.

[4] Cubitto M. A., Morán A. C., Commendatore M., Chiarello M. N., Baldini M. D., Siñeriz F. (2004). Effects of *Bacillus subtilis* O9 biosurfactant on the bioremediation of crude oil-polluted soils. *Biodegradation*.

[5] Van Hamme J. D., Singh A., Ward O. P. (2006). Physiological aspects. Part 1 in a series of papers devoted to surfactants in microbiology and biotechnology. *Biotechnology Advances*.

[6] Kiran S. G., Thomas A. T., Selvin J., Sabarathnam B., Lipton A. P. (2010). Optimization and characterization of a new lipopeptide biosurfactant produced by marine *Brevibacterium aureum* MSA13 in solid state culture. *Bioresource Technology*.

[7] Sivapathasekaran C., Mukherjee S., Ray A., Gupta A., Sen R. (2010). Artificial neural network modeling and genetic algorithm based medium optimization for the improved production of marine biosurfactant. *Bioresource Technology*.

[8] Satpute S. K., Banat I. M., Dhakephalkar P. K., Banpurkar A. G., Chopade B. A. (2010). Biosurfactants, bioemulsifiers and exopolysaccharides from marine microorganisms. *Biotechnology Advances*.

[9] Banat I. M., Makkar R. S., Cameotra S. S. (2000). Potential commercial applications of microbial surfactants. *Applied Microbiology and Biotechnology*.

[10] Ron E. Z., Rosenberg E. (2002). Biosurfactants and oil bioremediation. *Current Opinion in Biotechnology*.

[11] Franzetti A., Tamburini E., Banat I. M., Sen R. (2010). Applications of biological surface active compounds in remediation technologies. *Biosurfactants*.

[12] Mulligan C. N. (2005). Environmental applications for biosurfactants. *Environmental Pollution*.

[13] Parra J. P., Guinea J., Manresa M. A. (1989). Effect of the carbon source on biosurfactant production by *Pseudomonas aeruginosa* 44T1. *Journal of the American Chemical Society*.

[14] Lang S., Wullbrandt D. (1999). Rhamnose lipids—biosynthesis, microbial production and application potential. *Applied Microbiology and Biotechnology*.

[15] Easterling E. R., French W. T., Hernandez R., Licha M. (2009). The effect of glycerol as a sole and secondary substrate on the growth and fatty acid composition of Rhodotorula glutinis. *Bioresource Technology*.

[16] Sharma Y. C., Singh B., Upadhyay S. N. (2008). Advancements in development and characterization of biodiesel: a review. *Fuel*.

[17] da Silva G. P., Mack M., Contiero J. (2009). Glycerol: a promising and abundant carbon source for industrial microbiology. *Biotechnology Advances*.

[18] Morikawa M., Daido H., Takao T., Murata S., Shimonishi Y., Imanaka T. (1993). A new lipopeptide biosurfactant produced by *Arthrobacter* sp. strain MIS38. *Journal of Bacteriology*.

[19] Siegmund I., Wagner F. (1991). New method for detecting rhamnolipids excreted by *Pseudomonas* species during growth on mineral agar. *Biotechnology Techniques*.

[20] Koch A. K., Kappeli O., Fiechter A., Reiser J. (1991). Hydrocarbon assimilation and biosurfactant production in *Pseudomonas aeruginosa* mutants. *Journal of Bacteriology*.

[21] Zhang Y. M., Miller R. M. (1992). Enhanced octadecane dispersion and biodegradation by a *Pseudomonas* rhamnolipid surfactant (biosurfactant). *Applied and Environmental Microbiology*.

[22] Déziel E., Lépine F., Milot S., Villemur R. (2000). Mass spectrometry monitoring of rhamnolipids from a growing culture of *Pseudomonas aeruginosa* strain 57RP. *Biochimica et Biophysica Acta (BBA)—Molecular and Cell Biology of Lipids*.

[23] Leitermann F., Syldatk C., Hausmann R. (2008). Fast quantitative determination of microbial rhamnolipids from cultivation broths by ATR-FTIR Spectroscopy. *Journal of Biological Engineering*.

[24] Thavasi R. S., Sharm S., Jayalakshmi S. (2011). Evaluation of screening methods for the isolation of biosurfactant producing marine bacteria. *Journal of Petroleum & Environmental Biotechnology*.

[25] Santa-Anna L. M., Sebastian G. V., Menezes E. P. (2002). Production of biosurfactants from *Pseudomonas aeruginosa* PA1 isolated in oil environments. *Brazilian Journal of Chemical Engineering*.

[26] Prieto L. M., Michelon M., Burkert J. F. M., Kalil S. J., Burkert C. A. V. (2008). The production of rhamnolipid by a *Pseudomonas aeruginosa* strain isolated from a southern coastal zone in Brazil. *Chemosphere*.

[27] Mulligan C. N., Gibbs B. F. (1989). Correlation of nitrogen metabolism with biosurfactant production by *Pseudomonas aeruginosa*. *Applied and Environmental Microbiology*.

[28] Ishigami Y., Gama Y., Nagahora H., Yamaguchi M., Nakahara H., Kamata T. (1987). The pH-sensitive conversion of molecular aggregates of rhamnolipid biosurfactant. *Chemistry Letters*.

[29] Schenk T., Schuphan I., Schmidt B. (1995). High-performance liquid-chromatographic determination of the rhamnolipids produced by *Pseudomonas aeruginosa*. *Journal of Chromatography A*.

[30] Sabra W., Kim E.-J., Zeng A.-P. (2002). Physiological responses of *Pseudomonas aeruginosa* PAO1 to oxidative stress in controlled microaerobic and aerobic cultures. *Microbiology*.

[31] Medina G., Juárez K., Valderrama B., Soberón-Chávez G. (2003). Mechanism of *Pseudomonas aeruginosa* RhlR transcriptional regulation of the rhlAB promoter. *Journal of Bacteriology*.

[32] Aguirre-Ramírez M., Medina G., González-Valdez A., Grosso-Becerra V., Soberón-Chávez G. (2012). The *Pseudomonas aeruginosa rmlBDAC* operon, encoding dTDP-L-rhamnose biosynthetic enzymes, is regulated by the quorum-sensing transcriptional regulator RhlR and the alternative sigma factor *σ*
^S^. *Microbiology*.

[33] Schuster M., Lostroh C. P., Ogi T., Greenberg E. P. (2003). Identification, timing, and signal specificity of *Pseudomonas aeruginosa* quorum-controlled genes: a transcriptome analysis. *Journal of Bacteriology*.

[34] Müller M. M., Hörmann B., Syldatk C., Hausmann R. (2010). *Pseudomonas aeruginosa* PAO1 as a model for rhamnolipid production in bioreactor systems. *Applied Microbiology and Biotechnology*.

[35] Roy P. H., Tetu S. G., Larouche A. (2010). Complete genome sequence of the multiresistant taxonomic outlier *Pseudomonas aeruginosa* PA7. *PLoS ONE*.

